# Local War Committees and Local Authorities

**Published:** 1916-08-19

**Authors:** 


### LOCAL WAR COMMITTEES AND LOCAL AUTHORITIES.

THE demands made upon the medical profession by the Army authorities must in the very nature of things cause both much dislocation of professional work and a considerable degree of inconvenience to individual practitioners. To take away from their ordinary duties some thousands of doctors is a proceeding which produces inevitably a serious disturbance of existing arrangements, and this not only for the men who are taken but also for those who are left behind. The civil necessities of the general population continue whatever be the course of the war, and they can only be met by a redistribution of the work among the practitioners who remain at home. To meet this double request has meant an attempt to organise the whole of the medical profession, and this responsibility has been undertaken by machinery which has been set up for the purpose by the profession itself. Included in this machinery are local war committees whose duty it is to arrange the available doctors in such a fashion that, while the needs of the Army are supplied, the various districts of the country are not deprived to a dangerous degree of medical help and service. In a word, these committees have to decide in individual areas which doctors should take military service and which should remain at home; and in reference to the latter how the practices of their absent colleagues should be conducted.

The work involved in the arrangements here indicated has been, and still is, enormous, and it is being undertaken by voluntary services, which are none the less real because they do not reach the public eye. There are substantial complaints that such work is being hindered by official departments and by local authorities, which in certain districts and for reasons of their own upset the arrangements made by the local medical profession. As a result, a certain number of the younger men and of men free from family responsibilities are kept to official duties when, according to the local war committee, these duties could be efficiently discharged by seniors, while the juniors could be set free for military work. Such a position is one which calls for plain speaking. Either the judgment of the profession must be accepted or the profession must be freed from all responsibility in the matter. It is impossible to expect that practitioners who are voluntarily giving time and effort at the request of the authorities will continue to take all this trouble if their advice is ignored and pushed aside. Anxious as these men are to respond fully to every patriotic claim it is inevitable that they will be discouraged if secret arrangements made without their knowledge are to prevail over their carefully considered judgments. The authorities must not imagine that they will continue to receive the help and guidance which they have solicited if they ostentatiously disregard the advice given to them. They may either manage the whole of the business themselves or they may ask the medical profession to undertake it. But they cannot have the advantage of the latter course and at the same time act as though they themselves were the organisers of the enterprise. Between the one course and the other they must make their choice, and to it they must consistently adhere.

